# Thermal insulation and stability of polysiloxane foams containing hydroxyl-terminated polydimethylsiloxanes†

**DOI:** 10.1039/c8ra00222c

**Published:** 2018-03-08

**Authors:** Chunyu Zhang, Lijie Qu, Yingnan Wang, Tianlu Xu, Chunling Zhang

**Affiliations:** Key Laboratory of Automobile Materials, Ministry of Education, College of Materials Science and Engineering, Jilin University Changchun 130025 PR China clzhang@jlu.edu.cn +86 431 88498137 +86 431 85095170

## Abstract

An effective method was described here to improve the thermal insulation and stability of polysiloxane foam (SIF) by controlling the chain length of hydroxyl-terminated polydimethylsiloxane (OH-PDMS). A series of SIFs were prepared through foaming and cross-linking processes with different cross-linking densities. The morphology of SIF was investigated by environmental scanning electron microscopy. The results demonstrated that increasing the chain length of OH-PDMS reduced the average cell size from 932 μm to 220 μm. Cell density ranged from 4.92 × 10^6^ cells per cm^3^ to 1.64 × 10^8^ cells per cm^3^. The thermal insulation capability was significantly enhanced, and the SIF derived from the long-chain OH-PDMSs yielded a minimum thermal conductivity of 0.077 W mK^−1^. Cell size reduction and an increase in cell density were considered to be the main factors to reduce thermal conductivity. Thermal stability, which was also improved, mainly depended on the free motion rate of the polysiloxane chains and cross-linking density of the polysiloxane networks.

## Abstract 2

The thermal insulation and stability of polysiloxane foam was improved by an easy operating method.

## Graphical abstract

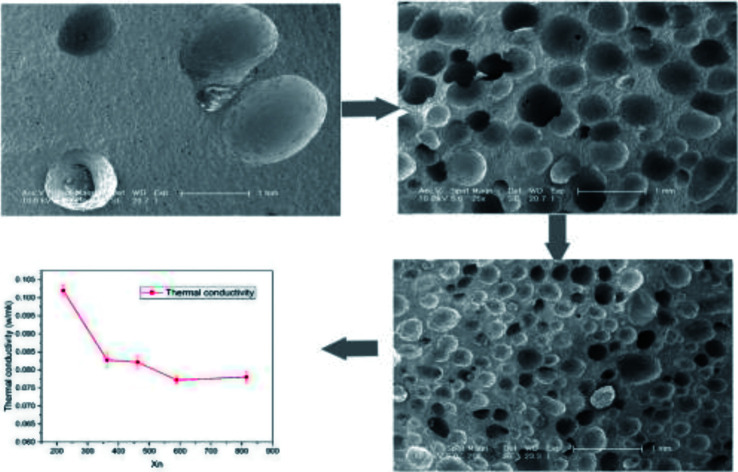

## 1. Introduction

Polymer foams have been attracting great interest because of their good properties, such as their light weight, low thermal conductivity, high specific surface area, and strong sound absorption.^1–7^ Polyurethane, polystyrene, polyethylene, polypropylene, and polyvinyl chloride are some of the commonly used conventional polymer foams.^8^ Polysiloxane foam (SIF) is a cellular polymer material with a backbone of siloxane bonds. The polymer backbone is “inorganic” in nature, while the substituted group on the silicon atom are generally “organic”.^9,10^ SIF materials exhibit many outstanding properties, such as high and low-temperature resistance, chemical stability, electrical resistance, and good biological compatibility because of their unique structure.^11^ Thus, SIFs have been widely used for aerospace-related materials, heat-resistant, and shock-absorbing materials.^12–14^

The thermal insulation and thermal stability of SIF materials have been improved by introducing functional organic or functional inorganic groups.^15–17^ Vedolotti *et al.* used sol–gel approach to fabricate a polyurethane foam with polysiloxane-domains to improve the thermal insulation capability of conventional foam material. Their results showed that hybrids exhibited a 22% reduction in thermal conductivity compared with pristine polyurethane foam.^18^ Chruściel and Leśniak used poly(diphenyl methane isocyanate) and carbofunctional polysiloxanes to modify polysiloxane by preparing a self extinguishing SIF at room temperature.^19^ The incorporated organic groups could participate in the reaction, such as the cross-linking reaction. Additional covalent bonds may be formed. Introducing functional organic groups in the polymeric foam is a direct method to combine the properties offered by these groups. Verdejo *et al.* studied the effects of functionalized graphene sheet and carbon nanotubes on the thermal properties of polysiloxane nanocomposites.^20,21^ The results showed that thermal stability could be enhanced at low mass fraction (0.5 wt%) of the fillers. We previously investigated the effects of two kinds of modified montmorillonite on the thermal stability and thermal insulation performance of SIF.^22^ We also explored the effects of hollow microspheres on the thermal insulation of SIF.^23^ The inorganic particles acted as the nuclear agent and offered a large number of nucleation sites, which was favorable in the formation of a uniform cell morphology. However, it easily aggregated in the polymer matrix. Almost all inorganic fillers improved the thermal stability of SIFs, but some of these fillers, such as functionalized graphene sheet and carbon nanotubes, exhibited a negative impact on the thermal insulation capability.

Polymeric foams were thermal insulation materials, the porous structure of the foam restrained the heat transfer and reduced the heat loss.^15^ Thermal conductivity is a vital heat transfer property of foam materials. Heat transfer generally involves solid heat conduction, gas heat conduction, thermal radiation, and heat convection between a solid and a gas. The microstructure and foam density are considered the main factors that influence heat transfer. Cell size reduction and increase in cell density are beneficial to reduce thermal conductivity. The polysiloxane foam materials exhibited a higher thermal stability than that of conventional polymer foam [9]. It may be obtained from the linear polysiloxanes containing active functional groups. Hydroxyl-terminated polydimethylsiloxanes (OH-PDMSs) are the basic component of an SIF material. It is usually synthesized by ring-opening polymerization of hexamethylcyclotrisiloxane or octamethylcyclotetrasiloxane (D_4_).^24–26^ Cross-linking is based on the polycondensation and polyaddition reaction in the presence of a catalyst. One of the processes occurs between the silanol groups and the Si–H groups of polymethylhydrosiloxane (PMHS) with the evolution of hydrogen. The balance between foaming and cross-linking was obtained by increasing the chain length of OH-PDMS. This process promoted the formation of enhanced cellular structure. The present work is performed to improve the thermal insulation and stability of SIF by controlling the chain length of OH-PDMS. This method is easy to operate and avoid the poor compatibility compared with the addition of inorganic particles into the polysiloxane matrix.

## 2. Experimental

### 2.1 Materials

Octamethylcyclotetrasiloxane (99.9%) was purchased from Shandong dayi Chemical Industrial Co., Ltd. (Jinan County of Shandong Province, China) and distilled over CaH_2_. Solid super acid was made in our laboratory. Petroleum ether was purchased from Aladdin Biological Technology Co., Ltd. (Shanghai, China). Vinyl-terminated PDMS (vinyl content ≥ 9.5%) was commercially obtained from Huazhirun Chemical Industrial Co., Ltd. (Shanghai, China). PMHS (hydrogen content 1.5–1.65%) was also was commercially obtained from Huazhirun Chemical Industrial Co., Ltd. (Shanghai, China). A high-performance platinum catalyst (2000 ppm) was supplied by Nuohai Chemical Plant (Guangzhou County of Guangdong Province, China). Foaming agent (YQ-100, the decomposition temperature is above 100 °C) was supplied by Yingquan Chemicals Industrial Co., Ltd. (Shenzhen County of Guangdong Province, China). Water used in this study was deionized in the laboratory.

### 2.2 Synthesis of the OH-PDMS

**Scheme 1:**
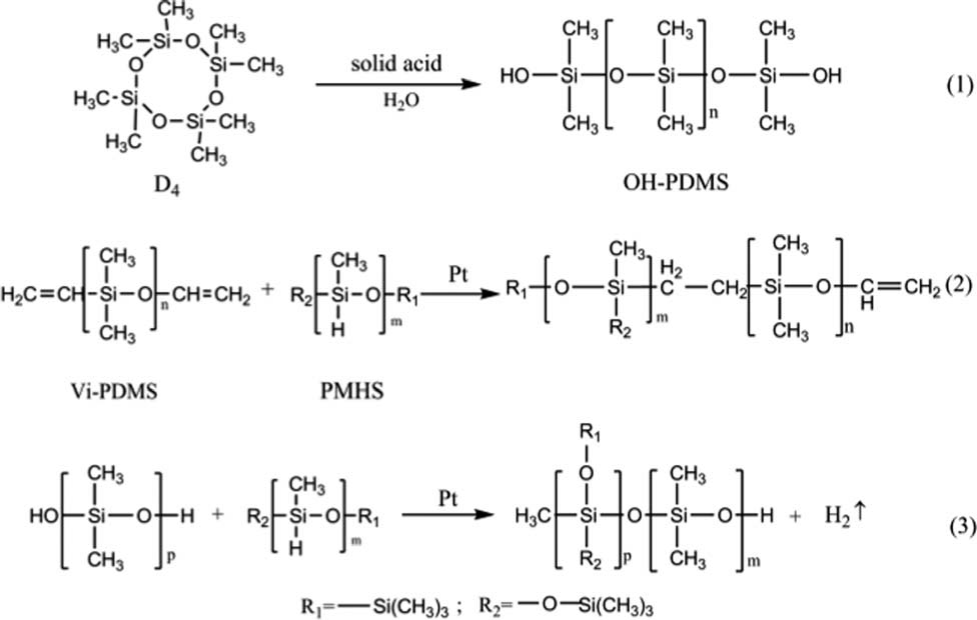
Preparation of hydroxyl-terminated polydimethylsiloxanes (OH-PDMS) and polysiloxane foam (SIF).

Octamethylcyclotetrasiloxane and petroleum ether were placed in a 250 mL three-necked, round-bottomed flask and magnetically stirred for 10 min. Then, the solid super acid was added, and deionized water was added to the reaction vessel as the final component after a period of time to terminate the reaction. The solid super acid was separated by filtration, and the remaining water and low-boiling residues were removed by a rotary evaporator. The molecular weight of OH-PDMS can be controlled by adjusting the reaction condition, such as reaction temperature, the amount of catalyst, the amount of end-blocking agent and the polymerization time. The detail result was showed in ESI (Tables P1, P2, P3 and P4).† The corresponding preparation scheme of OH-PDMS is shown in Scheme 1(1).

### 2.3 Synthesis of the SIF

**Fig. 1:**
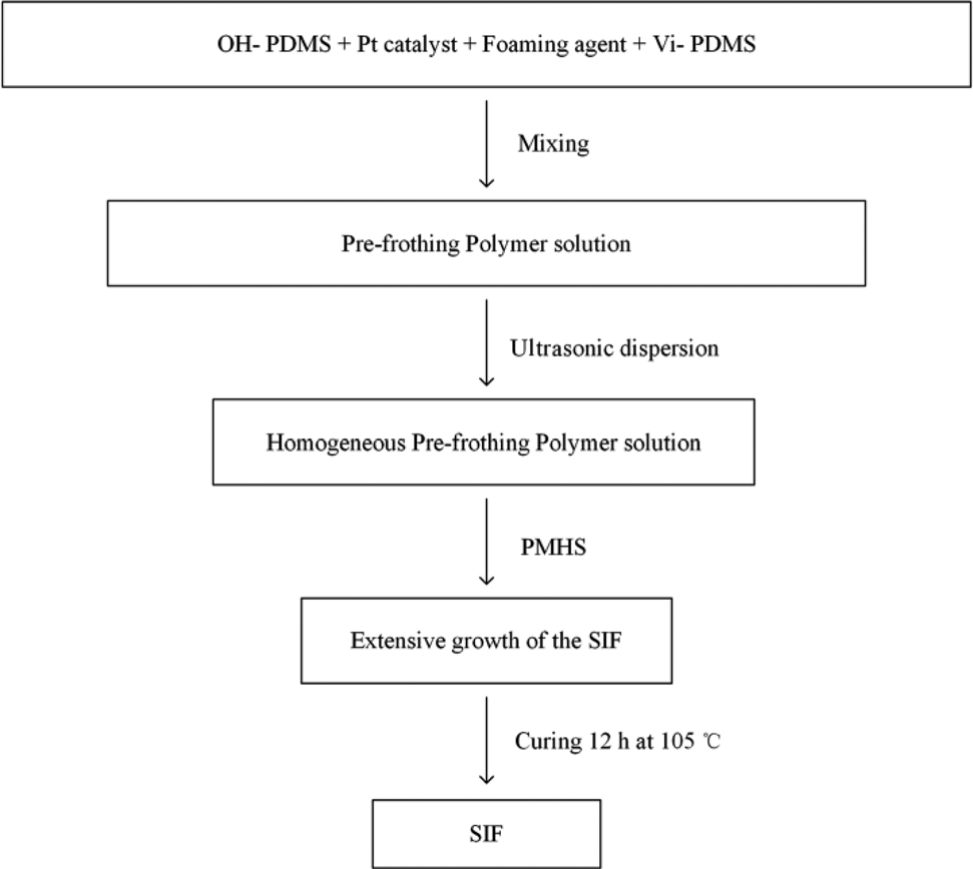
Flowchart for preparation of polysiloxane foam.

25 g of OH-PDMS was added to a vessel, then, the high-performance platinum catalyst were added, next, the other additives, including the vinyl-terminated PDMS and the 1 g of foaming agent YQ-100 were added in the vessel. After each substance was added, the mixture was ultrasonically dispersed for 30 min. 5 g of PMHS was added to the reaction mixture as the final component. After carefully mixing all components, we observed the extensive growth of the SIF within 5–10 min. Subsequently, the samples were transferred into an oven to react completely at 105 °C. The five different chain length of OH-PDMS was used as the matrix to synthesize polysiloxane foam, which are recorded as S1, S2, S3, S4, S5 respectively. The preparation scheme is shown in Scheme 1(2 and 3), and the process of preparation is shown in Fig. 1.

### 2.4 Characterization

Fourier transform infrared (FTIR) spectra were obtained with a FTIR spectrophotometer (Tensor 27). The spectra of D_4_ and PDMSs were obtained by potassium bromide pellet technique, while the spectra of the SIFs were recorded through attenuated total reflectance. The molecular weight of the OH-PDMSs were investigated by gel permeation chromatography (GPC) measurements on a Waters breeze instrument using tetrahydrofuran as the eluent, the average degree of polymerization (*X*_n_) was used to characterize the length of the polysiloxane chains. The swelling degree of samples was measured by equilibrium swelling method. Equilibrium swelling degree was expressed as the ratio:*Q* = (*m* − *m*_0_)/*m*_0_ × 100%where *m*_0_ and *m* are the weights of the initial and swollen samples, respectively. The morphological of the samples in the rising foam direction were observed with an environmental scanning electron microscopy (SEM) system (XL30 ESEM FEG, FEI, China) at an accelerating voltage of 10 kV. The average cell size and cell distribution were calculated by the software for SEM image analysis (Nano Measurer System, 1.2.5).^27^ Cell density was defined as follows.^8,28^
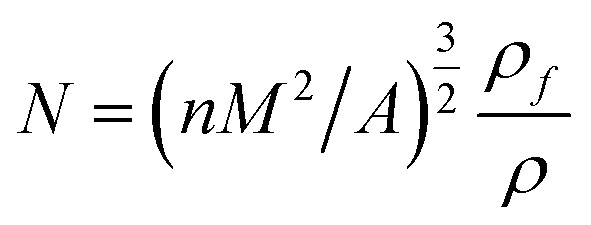
where *n* is the number of cells in the micrograph of the foam sample, *A* is the area of the micrograph (cm^2^), *ρ* is the density of polysiloxane matrix, *ρ*_f_ is the density of SIFs, and *M* is the magnification factor of the micrograph. Thermal conductivity was measured on a TPS 2000 hot-disk analyzer through a transient plane heat source method. The glass-transition temperature (*T*_g_) of the samples were investigated by differential scanning calorimetry (DSC) (TA Q20) analysis. Thermo-gravimetric analysis (TGA) of the samples were performed using a Pyris TGA (PerkinElmer) thermal analyzer with a heating rate of 10 °C min^−1^ from 50 °C to 800 °C under air and nitrogen atmospheres. Limiting oxygen index (LOI) was measured using a JF-3 type oxygen index apparatus (FESTEC, South Korea). The mechanical properties of SIF samples were measured on a universal testing machine (RGT-X010, China) at a crosshead speed of 5 mm min^−1^. The apparent foam density was expressed as the weight of the samples divided by its volume.

## 3. Results and discussion

### 3.1 Chemical characterization of SIF

**Fig. 2:**
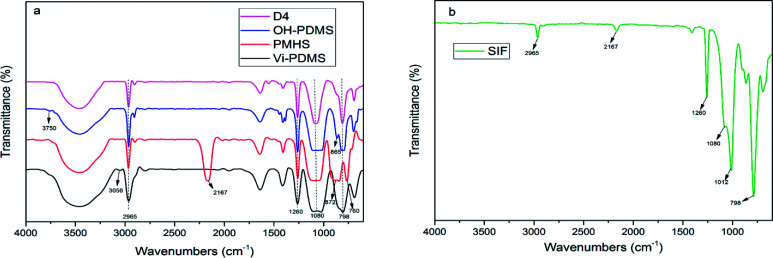
Fourier transform infrared spectrum of (a) PDMS, (b) SIF.

Fig. 2 shows the spectra of PDMS and SIF. The peak at 2167 cm^−1^ was the absorption of Si–H group. The peak at 3056 cm^−1^ was the absorption of =C–H stretching vibration. The peak at 3750 cm^−1^ was the absorption of Si–OH deformation vibration. The peak at 1260 cm^−1^ and 2965 cm^−1^ was the absorption of Si–CH_3_ bending vibration and stretching vibration, respectively. The absorptions for double bonds and silanol groups were clear in the Fig. 2(a), but it was not appeared in the Fig. 2(b). This result illustrates that SIF was successfully synthesized.

### 3.2 Cross-linking density of the polysiloxane network

**Fig. 3:**
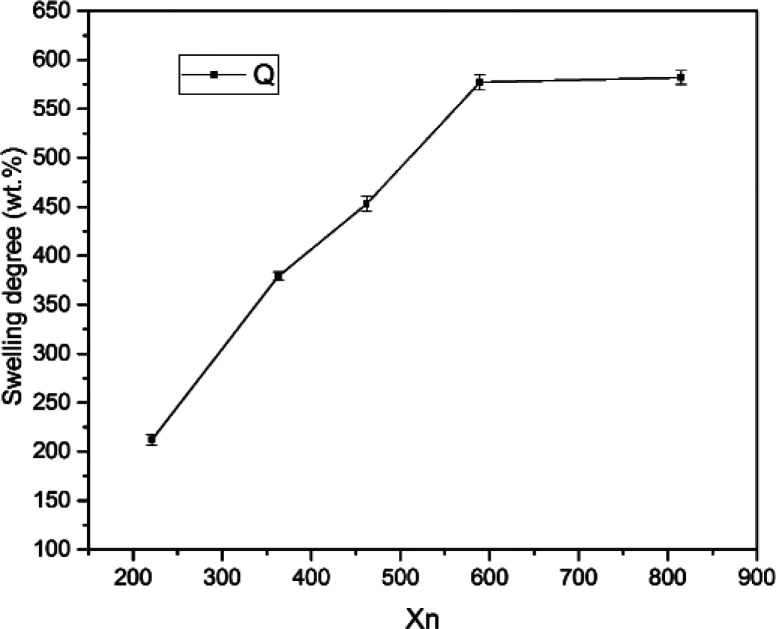
The swelling degree of SIFs.

Fig. 3 shows the swelling degree of SIFs with different chain lengths of OH-PDMS. Polysiloxane networks were obtained by polycondensation and polyaddition reaction. The result of equilibrium swelling showed that the chain length of the applied OH-PDMS strongly affected the cross-linking density of the prepared system. As can be seen from Table 1, the swelling degree of the long chain OH-PDMS derived networks were significantly higher, and cross-linking density was lower than that of the network prepared from the short-chain OH-PDMS.^29^ This phenomenon was attributed to the lower content of silanol groups of long-chain OH-PDMS. More silanol groups indicated more cross-linking points. The polycondensation reaction between the silanol and Si–H groups was closely associated with the content and reactivity of the silanol groups. The higher silanol and the free motion rate of the short chain OH-PDMS were the main factors for the greater cross-linking density.

**Table 1:** Properties of SIFs with different chain length of OH-PDMS

Sample code	*M*_n_	*M*_w_	PDI	*X*_n_	Swelling degree (%)	Cell size (μm)	Cell density (10^8^ cells per cm^3^)	Thermal conductivity (W mK^−1^)
S1	16 335	27 835	1.70	221	210 ± 6	932	0.0429 ± 0.0063	0.10200 ± 0.00162
S2	26 861	37 337	1.39	363	379 ± 4	480	0.2710 ± 0.0316	0.08276 ± 0.00210
S3	34 176	45 178	1.34	462	443 ± 8	340	0.3900 ± 0.0296	0.08215 ± 0.00175
S4	43 579	57 524	1.32	589	565 ± 9	220	1.6000 ± 0.0503	0.07720 ± 0.00115
S5	60 318	77 805	1.29	815	582 ± 7	240	1.6400 ± 0.0757	0.07800 ± 0.00156

### 3.3 Morphological analysis

**Fig. 4:**
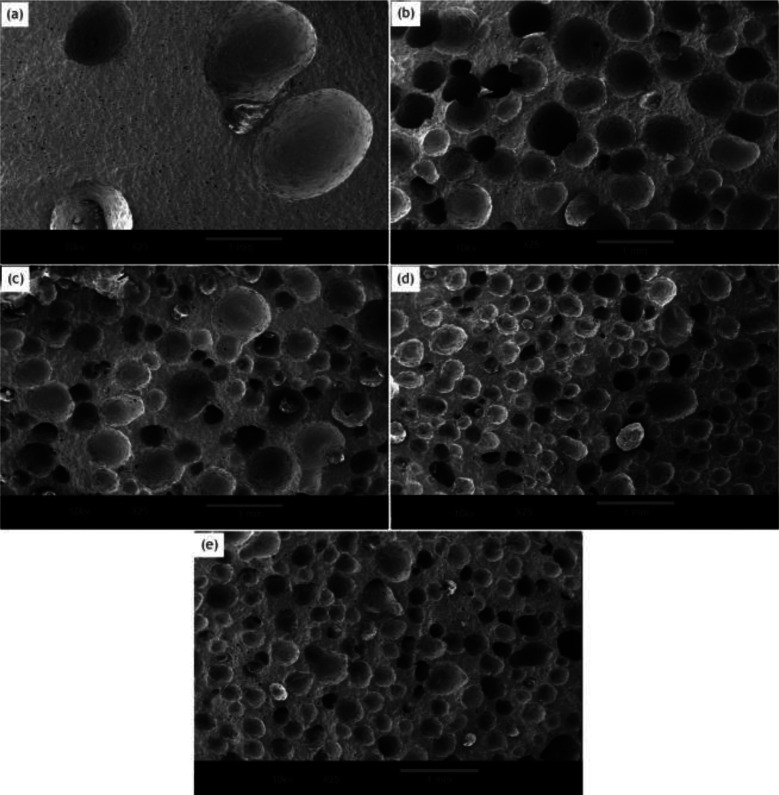
Effect of the chain length of OH-PDMS on the cell morphology in SIFs: (a) S1, (b) S2, (c) S3, (d) S4, and (e) S5.

**Fig. 5:**
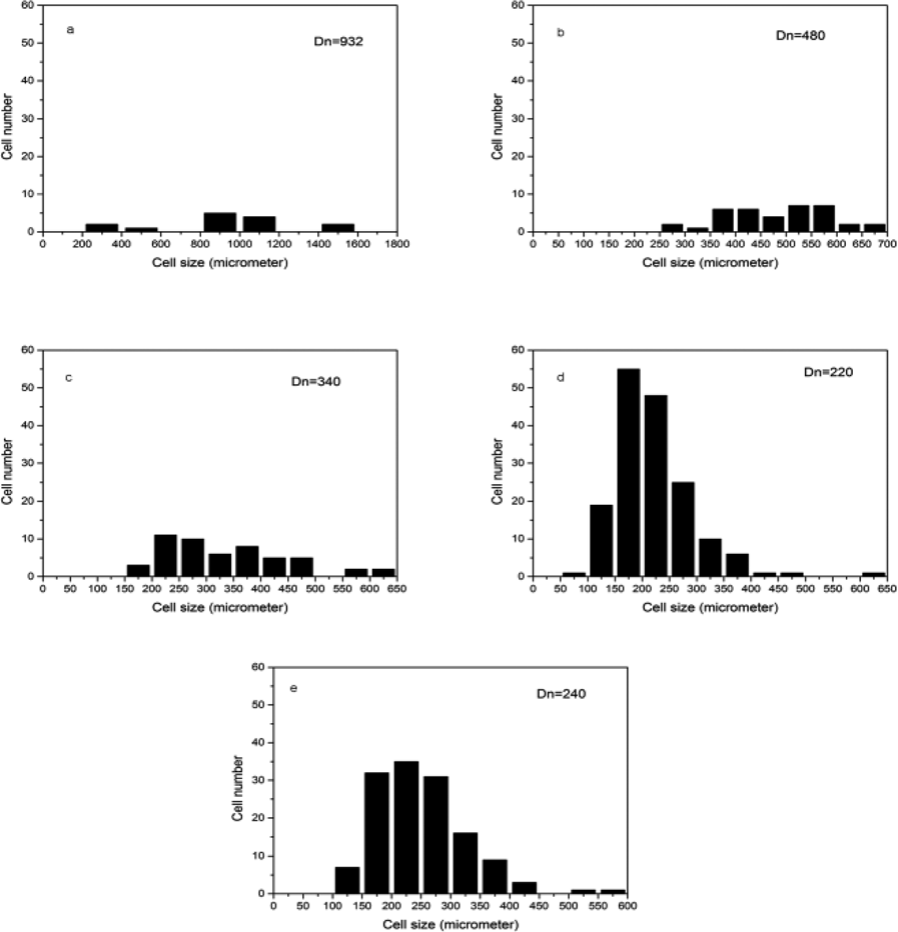
Cell distribution of SIFs with different chain lengths of OH-PDMS obtained from the SEM images: (a) S1, (b) S2, (c) S3, (d) S4, and (e) S5.

**Fig. 6:**
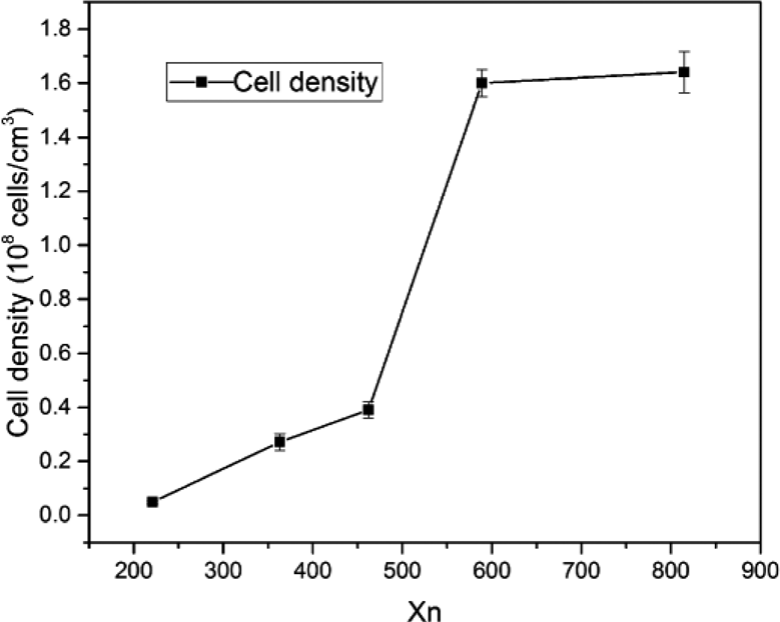
Cell density of SIFs.

Fig. 4 shows the cell morphology of the SIFs with different chain lengths of OH-PDMS. SEM investigations showed that increasing the chain length of OH-PDMS enhanced the cell number and cell density but reduced the cell size. The average cell size and cell density were calculated by the software for SEM image analysis (Nano Measurer System, 1.2.5). Fig. 5 shows that the cell sizes ranged from 90 μm to 1500 μm, and the average diameter ranged from 220 μm to 932 μm. The cell density ranged from 4.92 × 10^6^ cells per cm^3^ to 1.64 × 10^8^ cells per cm^3^ (Fig. 6), which was raised by nearly two orders of magnitude. The peak in the cell size distribution curve in the S1 sample was located at 900 μm but it shifted to a lower value when the chain length of the OH-PDMS was increased (*i.e.*, 525 μm for S2). These cell structural changes are related to the cross-linking density of the polysiloxane network and viscosity of the system. The higher cross-linking density may accompany a dramatic reaction process, but lower cross-linking density indicated a mild reaction process, which is beneficial to form higher cell density and uniform cell distribution. The higher reactivity and free motion rate of short chain OH-PDMS led to the greater speed of foaming reaction than that of the cross-linking reaction. The gas from the polycondensation escaped quickly in the reaction. All bubbles bursted in the polysiloxane matrix. This led to the lower cell density and the higher average cell size. The speed of the foaming and cross-linking reactions tended to be in equilibrium by increasing the chain length of OH-PDMS. This scenario was beneficial to the formation of an improved microstructure. Moreover, when a short-chain OH-PDMS was incorporated, the viscosity of the system was low, and the polysiloxane matrix showed good mobility. In this case, the microcell grew faster under the influence of gas pressure, resulting in larger cell size.^15^ The viscosity of the system significantly increased with the chain length of OH-PDMS, and the resistance of bubble growth also increased. These characteristics are conducive to form smaller cell size and uniform cell distribution. However, excessive viscosity may adversely affect the microstructure.

### 3.4 Thermal conductivity

**Fig. 7:**
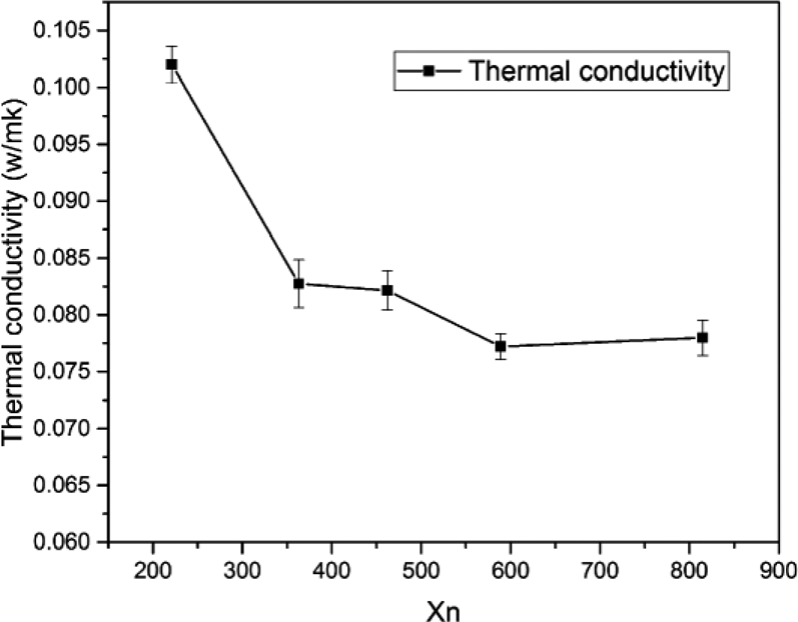
Thermal conductivity of SIFs with different chain lengths of OH-PDMS.

Thermal conductivity is an important parameter to characterize the thermal insulation capability of foam materials. Fig. 7 shows that the thermal conductivity ranged from 0.0772 W mK^−1^ to 0.102 W mK^−1^. The thermal conductivity decreased by increasing chain length of OH-PDMS up to a reduction of 24.5% for S4 compared with S1. The heat transfer of the polymer foam was jointly influenced by solid conduction, gas conduction, and radiation. Heat was conducted by the vibration of the atoms and molecules. The distance between gas molecules is larger than those of solid molecules. Thus, collisions between the gas molecules are not as violent as those in solids, resulting in the fastest and slowest heat conductions in solids, and gases, respectively.^30,31^ However, the heat transfer process of foam material was very complicated, our findings of thermal conductivity was directly related to their density and microstructure. The SIF with short-chain OH-PDMS exhibited lower cell density and larger cell size. Heat transfer in this sample was equivalent to that of the nonfoamed polysiloxane matrix, and this process is close to the continuous heat transfer in the solid phase. The SIF prepared from long chain OH-PDMS exhibited the better microstructure, such as the smaller cell size and higher cell density, which contributed to lower the heat transfer within the solid cell wall for increasing the contribution ratio of gases conduction to the total thermal conductivity. In addition, it interrupted the continuously solid conductivity through the cell wall and struts. Thus, the thermal conductivity showed a downward trend. The open cells should contribute to increase the thermal conductivity, but the closed cell expressed the opposite influence. This result is consistent with previous report, in which the cell content influenced the thermal conductivity to a lesser extent than the cell size.^18^ This phenomenon explains the slightly higher cell density but higher thermal conductivity of S5 than those of S4. The reduction in foam density decreased the radiation and solid phase conduction.^31^ This was also beneficial to decrease the thermal conductivity.

### 3.5 Thermal performance

**Fig. 8:**
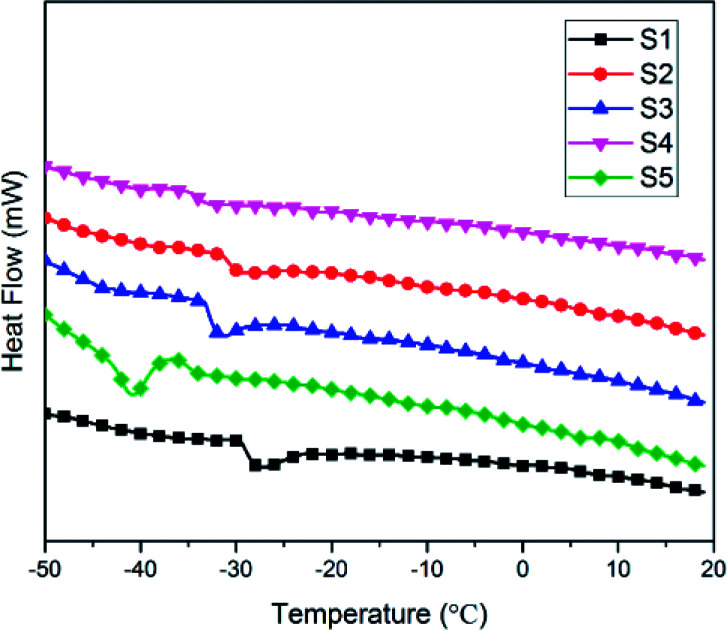
Differential scanning calorimetry curves of SIFs with different chain lengths of OH-PDMS.

Low-temperature resistance is one of the most important properties of polysiloxane, because this characteristic establishes the range of service temperature for silicone-based functional materials.^38^ The *T*_g_ is an important parameter of the thermal property of SIFs, which are only used at temperatures above *T*_g_. Fig. 8 shows the differential scanning calorimetry curves of the SIFs. The *T*_g_ decreased with increase in chain length of OH-PDMS. For example, S1 yielded a maximum *T*_g_ of −27.5 °C, while S5 had minimum *T*_g_ of −40.8 °C. Glass transition is generally ascribed to the segmental motion of the polymeric network, and *T*_g_ is determined by the degree of freedom for the segmental motion, cross-linking, and entanglement constrains.^32–34^ In this work, *T*_g_ mainly depended on the cross-linking density and the motion of polysiloxane chain. The SIFs prepared from the long-chain OH-PDMS showed lower cross-linking density and, consequently, lower *T*_g_. Moreover, the mobility of long-chain polysiloxane was more difficult than that of short chain. This characteristic was also beneficial to decrease the *T*_g_.

**Fig. 9:**
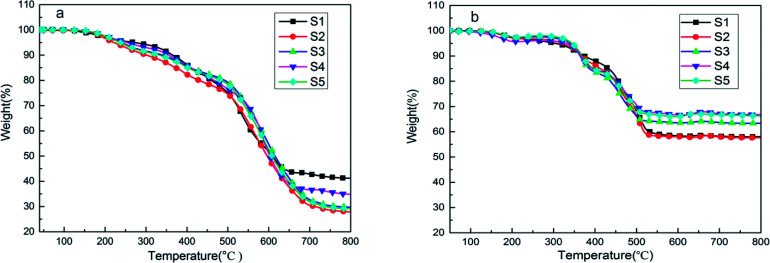
TGA curves of SIFs with different chain lengths of OH-PDMS under (a) nitrogen atmosphere and (b) air.

Fig. 9 shows the thermal decomposition behavior of SIFs under nitrogen atmosphere and air. Three degradation steps can be observed in the thermogram of SIFs under nitrogen atmosphere. The first degradation step occurred in the range of 150–300 °C. This phenomenon is due to the degradation of completely decomposed residual of foaming agent and the residual cyclosiloxane small molecule in the synthesis of PDMS. The second degradation step occurred at 300–450 °C, which could be ascribed to the scission of PMHS chain from the polysiloxane network.^35,36^ The PMHS was used as crosslinker, as mentioned above (Fig. 1), and the Si–H bond was excessive during the preparation of SIF. The Si–O bond exhibited substantial ionic and partially double bond characteristics, which are beneficial for the rearrangement reaction.^8^ The decomposition products may be cyclotetrasiloxane that contains the Si–H bond, which was the most easily formed stable cyclosiloxane. The third degradation step was observed in the temperature range 460–700 °C. This step was the major decomposition of the polysiloxane network and attributed to the Si-containing bonds redistribution reaction in the polysiloxane network. This reaction occurred at the intermolecular or intramolecular and accompanied by the evolution of volatile Si compound, which may be higher order cyclic siloxane.^29,35^ Another degradation mechanism could be attributed to the random scission, the high temperature increased the free motion rate of the polysiloxane chain and promoted the random degradation.

Fig. 9(b) shows the thermogram of SIFs under air. Three degradation steps and the similar temperature of the first two stages were also observed. These temperatures were in the range 140–230 °C and 240–420 °C, respectively. The first stage could be ascribed to the oxygenolysis of cyclosiloxane small molecule and completely decomposed residual of the foaming agent. The second degradation step may be attributed to the oxygenolysis of polysiloxane network, which may contain the oxidation of side chains and pyrolysis of substituent with the evolution of H_2_O and CO_2_. The third stage took place in the temperature range of 420–550 °C. During oxygen catalyzing decomposition, the radical derived from the second degradation stage may cross-link with each other. Thus, the main decomposition product was the black stable residual, but its specific composition was difficult to determine. This phenomenon explained the greater residual in air greater than in nitrogen. The oxygenolysis of the polysiloxane network is complex, and the catalyst (platinum) could form active centers to catalyze the decomposition of the polysiloxane network during depolymerization. Meanwhile, the activation energy of this catalytic degradation was lower than that of the non-catalytic reaction.^37–40^

As summarized in Table 2, *T*_5%_ is defined as the onset degradation temperature of SIFs. The S1 sample had higher temperature of initial decomposition and residual mass in nitrogen, because it had a higher cross-linking density. However, this sample showed a very small cell density, indicating the unsuccessful formation of SIF, the product was likely silicone rubber. Thus, S1 was excluded. The S4 sample showed higher onset degradation temperature and residual mass than the other samples, while S3 exhibited the highest temperature of maximum mass loss. The lower cross-linking density of the polysiloxane network, the possibility of intermolecular rearrangement is smaller. Hence, the SIFs prepared from long-chain OH-PDMSs showed higher temperature of maximum mass loss. Meanwhile, the long chain showed lower free motion rate, which negatively influenced the intramolecular rearrangement reaction. These results illustrated that SIFs derived from the long-chain OH-PDMS exhibited better thermal stability under nitrogen. However, they showed a different thermal behavior under air. The S1 sample exhibited the lowest onset degradation temperature but the highest temperature of maximum mass loss. The S4 sample had the highest residual mass of 66.7% at 800 °C. The cross-linking of polysiloxane could prevent the chain scission, the higher cross-linking density restrained the formation of radial. Thus, the SIFs prepared from the short-chain OH-PDMS exhibited higher temperature of maximum mass loss. However, the polysiloxane network prepared from the long-chain OH-PDMS may easily produce the radical. This characteristic is beneficial to the cross-linking reaction in the third degradation stage, which may be the reason for the higher residual mass of S4.

**Table 2:** Thermal properties of the SIFs

Sample code	*T*_g_ (°C)	*T*_5%_ (°C)		*T*_10%_ (°C)		Temperature of maximum mass (°C)		Residual mass at 800 °C (wt%)	
		Nitrogen	Air	Nitrogen	Air	Nitrogen	Air	Nitrogen	Air
S1	−27.5	272.2	304.6	365.2	374.1	532.3	512.2	41.3	58.0
S2	−31.3	221.7	322.8	301.4	369.0	583.5	505.3	27.9	57.6
S3	−32.5	238.7	338.4	341.0	364.4	595.8	454.3	29.7	63.3
S4	−34.4	253.6	328.2	356.0	365.4	586.8	466.3	34.7	66.7
S5	−40.8	237.2	344.2	333.1	368.9	590.5	463.2	29.1	66.2

### 3.6 Flame retardancy

**Fig. 10:**
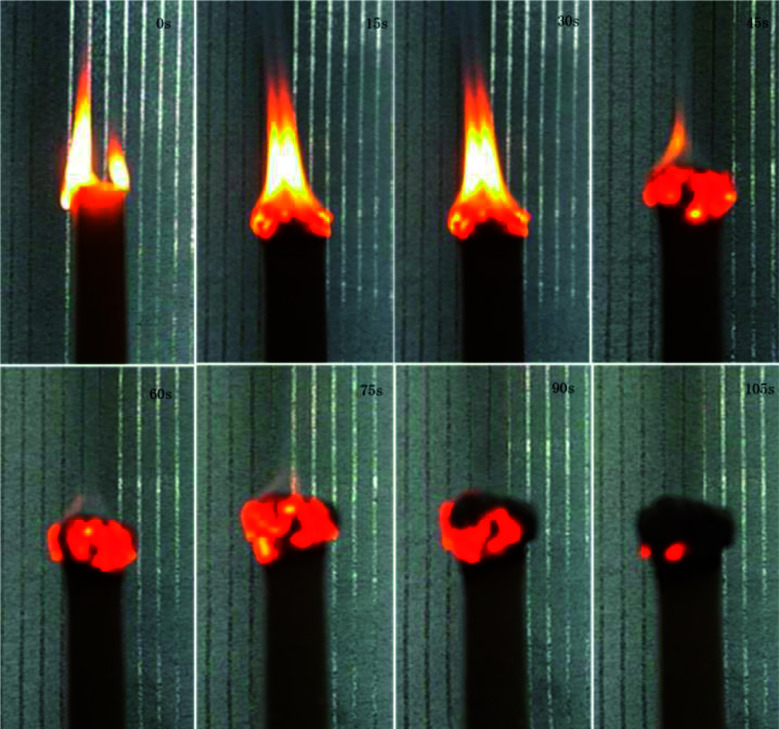
Combustion process of S4 sample under air.

**Fig. 11:**
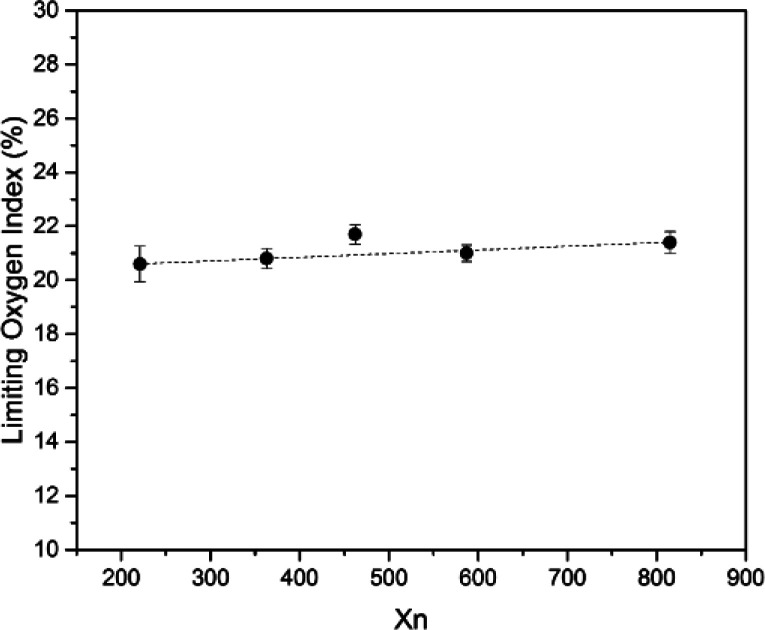
Limiting oxygen index of SIFs with different chain lengths of OH-PDMS.

SIF exhibited a certain degree of flame retardancy compared with conventional polymer systems.^41–44^ This characteristic is due to the fact that the combustion product of silicone-based polymers is silica but that of carbon-based polymers is carbon dioxide. Fig. 10 shows the burning process of S4 under air. The whole combustion process lasted for approximately 100 s. At the end of combustion, an off-white char layer was observed on the surface of the samples. This layer may act as a protective layer to prevent the spreading of flame, but the char is fragile. Fig. 11 shows the limiting oxygen index of SIFs. The limiting oxygen index measures the minimum oxygen concentration of the flowing gas mixed by nitrogen and oxygen required supporting downward flame combustion. This index could be used to evaluate the flame retardancy of the foam materials. The limiting oxygen index of SIFs samples showed an unremarkable increasing tendency. S3 yielded a maximum limiting oxygen index of 21.7%, while the rest of the samples had limiting oxygen index values of approximately 21%. This result is consistent with those obtained in a previous study.^20^

### 3.7 Mechanical properties

**Fig. 12:**
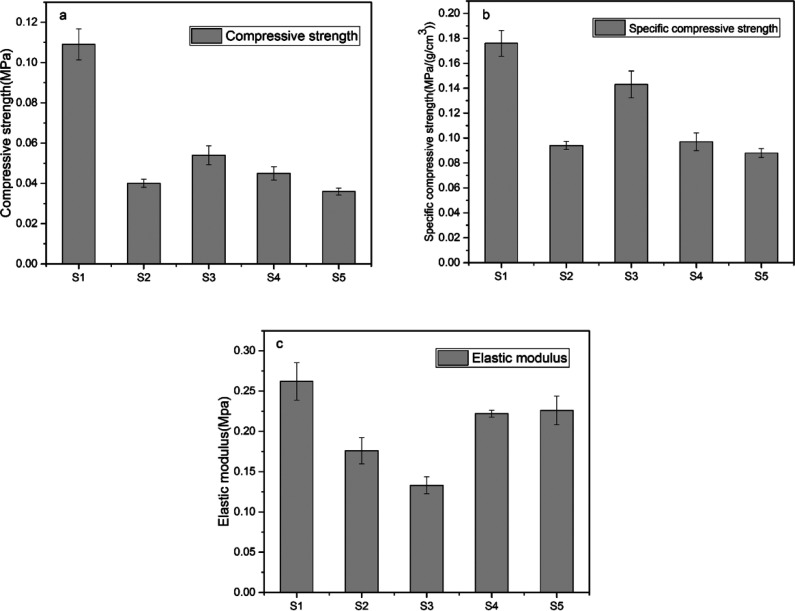
Mechanical strength of SIFs.

The compressive strength, specific compression strength, and elastic modulus were used to evaluate the mechanical property of the SIFs. Fig. 12 shows that S1 had highest compressive strength, specific compressive strength, and elastic modulus. However, this sample was considered to be an unsuccessfully foamed SIF. Its mechanical strength is consistent with an unfoamed polysiloxane elastomer. Thus, S1 was excluded, while S3 had the superior compression strength than the others.

Table 3 shows that the peak mechanical strengths of S3 were 0.054 MPa and 0.143 MPa (g cm^−3^)^−1^, while S5 exhibited higher elastic modulus of 0.227 MPa. Several factors influenced the mechanical strength of the foams.^45^ On the one hand, foam density is the main factor that determined the mechanical behavior of cellular materials. On the other hand, mechanical properties are also influenced by the characteristics of the microstructure.^15,46^ The S3 sample yielded a minimum foam density. Thus, it exhibited higher compressive strength and specific compression strength than the other samples. Smaller cell size implies a relatively stiffer bubble, and the homogeneous cell size distributions may lead to an increase in mechanical properties. These results illustrated that the SIFs derived from long-chain OH-PDMSs exhibited smaller cell size and narrower cell distribution, which may establish the higher elastic modulus.

**Table 3:** Compressive strength, specific compressive strength, and elastic modulus of SIFs

Sample code	Foam density (g cm^−3^)	Compressive strength (MPa)	Specific compressive strength (MPa (g cm^−3^)^−1^)	Elastic modulus (MPa)
S1	0.6109 ± 0.0075	0.1091 ± 0.0077	0.1762 ± 0.0103	0.2621 ± 0.0231
S2	0.4255 ± 0.0090	0.0402 ± 0.0020	0.0943 ± 0.0032	0.1768 ± 0.0164
S3	0.3805 ± 0.0132	0.0542 ± 0.0047	0.1432 ± 0.0108	0.1331 ± 0.0105
S4	0.4252 ± 0.0037	0.0450 ± 0.0033	0.0974 ± 0.0070	0.2223 ± 0.0044
S5	0.4170 ± 0.0025	0.0364 ± 0.0017	0.0886 ± 0.0037	0.2267 ± 0.0179

## 4. Conclusions

The present work presented a method for improving the thermal stability and thermal insulation of polysiloxane foam by regulating the chain length of OH-PDMS. A series of SIF was prepared through foaming and cross-linking processes with different cross-linking densities. The balance between foaming and cross-linking was beneficial to produce the uniform cellular structure. The increase in chain length of OH-PDMS accompanied the increase in viscosity of the system, which also contributed to the change in the microstructure.

The SIF prepared from the long-chain OH-PDMS displayed lower thermal conductivity than the short-chain products. The microstructure and foam density of the foam were both considered as factors that influenced the thermal conductivity of SIFs. However, cell microstructure was the main factor. The higher cell density and smaller cell size enhanced the contribution ratio of gases conduction to the total thermal conductivity. In addition, it interrupted the continuous solid conductivity through the cell wall and struts. Thermal stability was also enhanced by increasing the chain length of OH-PDMS. Thermo-gravimetric analysis results indicated that SIFs possessed distinct decomposition mechanisms under nitrogen and air. The *T*_g_ decreased to −40.8 °C, which indicated that the low-temperature resistance of SIFs was enhanced. Although the silicone-based polymer exhibited a certain degree of flame retardancy, no significant improvement in flame retardancy was observed when the chain length of OH-PDMS was adjusted. The lower foam density and homogeneous cellular structure were beneficial to improve mechanical property.

## Conflicts of interest

There are no conflicts to declare.

## Supplementary Material

RA-008-C8RA00222C-s001
