# Supplementary material for: Thermal insulation and stability of polysiloxane foams containing hydroxyl-terminated polydimethylsiloxanes
Source: RSC Adv. 2018 Mar 8;8(18):9901–9. doi: 10.1039/c8ra00222c (PMC9078707; doi:10.1039/c8ra00222c)
Supplement: RA-008-C8RA00222C-s001 [file RA-008-C8RA00222C-s001.pdf]

Electronic Supplementary Material (ESI) for RSC Advances.
This journal is © The Royal Society of Chemistry 2018

The molecular weight of OH-PDMS can be controlled by adjusting the reaction condition, such as reaction temperature, the amount of catalyst, the amount of end-blocking agent and the polymerization time. In the reaction process, the mass of octamethylcyclotetrasiloxane was 40g.

Table P1. Effect of reaction temperature on the molecular weight of OH-PDMS.

Run	Temperature (℃)	Mn	Mw	PDI
1	70	29535	40201	1.36
2	80	31143	41042	1.32
3	90	39790	69945	1.76
4	100	40224	64760	1.61
5	120	47210	108583	2.30

Conditions: The polymerization was carried for 6 h, the amount of catalyst was 0.4g, and the amount of end-blocking agent was 0.0486g.

Table P2. Effect of the amount of catalyst on the molecular weight of OH-PDMS.

Run	Catalyst (g)	Mn	Mw	PDI
1	0.2	22312	29643	1.32
2	0.4	26608	36985	1.39
3	0.6	45235	67169	1.48
4	0.8	60318	77850	1.28
5	1.0	63478	91997	1.44

Conditions: The polymerization was carried for 6 h, the reaction temperature was 80℃, and the amount of end-blocking agent was 0.0486g.

Table P3. Effect of the amount of end-blocking agent on the molecular weight of OH-PDMS.

Run	end-blocking agent (g)	Mn	Mw	PDI
1	0.0810	20136	26378	1.31
2	0.0607	26718	36870	1.38
3	0.0486	30691	39898	1.30
4	0.0405	40182	56656	1.41
5	0.0347	46286	62948	1.36

Conditions: The polymerization was carried for 6 h, the reaction temperature was 80℃, and the amount of catalyst was 0.4g.

Table P4. Effect of the polymerization time on the molecular weight of OH-PDMS.

Run	polymerization time (h)	Mn	Mw	PDI
1	3	13338	22407	1.70
2	4	16335	27769	1.68
3	5	25306	39224	1.55
4	6	28674	42437	1.48
5	7	31982	45094	1.41

Conditions: The reaction temperature was 80℃, and the amount of catalyst was 0.4g, and the amount of end-blocking agent was 0.0486g.
